# Dietary prebiotic-stevioside modulates the growth, antioxidant enzymes, and immune response in thinlip mullets (*Liza ramada*) subjected to chronic cold stress

**DOI:** 10.1186/s12917-025-04814-9

**Published:** 2025-05-21

**Authors:** Akram Ismael Shehata, Mohammed F. El Basuini, Ayaat M. Elmaghraby, Mayada Alhoshy, Ali A. Soliman, Asem A. Amer, Nermin A. Ibrahim, Yusuf Jibril Habib, Mahmoud S. Gewaily, Islam I. Teiba, Shimaa A. Shahin

**Affiliations:** 1 Department of Animal and Fish Production, Faculty of Agriculture (Saba Basha), Alexandria University, Alexandria, 21531 Egypt; 2 Faculty of Agriculture, Tanta University, Tanta, 31527 Egypt; 3 Faculty of Desert Agriculture, King Salman International University, El Tor, South Sinai Egypt; 4 Nucleic Acids Research Department, Genetic Engineering and Biotechnology Research Institute (GEBRI), City of Scientific Research and Technological Applications, Alexandria, Egypt; 5 Independent Researcher, Alexandria, 21648 Egypt; 6 Fish Nutrition Laboratory, Aquaculture Division, National Institute of Oceanography and Fisheries, Alexandria, 21556 Egypt; 7 Department of Fish Nutrition and Feed Technology, Central Laboratory for Aquaculture Research, Agricultural Research Center, Abbassa, Abo-Hammad, Sharqia, 44662 Egypt; 8 Laboratory of Genetics, Aquaculture Division, National Institute of Oceanography and Fisheries (NIOF), Cairo, Egypt; 9 Medical Analysis Department, Tishk International University, Erbil, Iraq; 10 Department of Anatomy and Embryology, Faculty of Veterinary Medicine, Kafrelsheikh University, Kafr El-Sheikh, 33516 Egypt

**Keywords:** Gene expression, Low temperature stress, Stevioside, Thinlip mullet

## Abstract

Plant active ingredients, including stevioside, with their prebiotic properties and antioxidant potential, hold promise as innovative components in aquaculture. This study assessed the multi-level impacts of stevioside (0, 100, 200, 300, 400, or 500 mg/kg) on thinlip mullet (N = 540 juveniles with an initial weight 3.50 ± 0.07 g) exposed to a long-term low temperature regime (18 °C) for 60 days. Growth, blood parameters, enzymes, immune genes, and histological structure of intestine and liver were analyzed. Moderate stevioside doses (300–500 mg/kg) significantly boosted growth, with 400 and 500 mg/kg groups showing the highest final and daily weight gain. These doses also increased total protein in blood serum. Interestingly, stevioside did not affect key blood markers like albumin, triglycerides, liver enzymes (ALT and AST), urea, or creatinine. However, it markedly enhanced (*P* < 0.05) antioxidant enzyme activity in liver (SOD, CAT, GPx) of experimental groups compared to the control group. Gene expression analysis in liver tissue revealed notable effects. Lower stevioside doses (100–300 mg/kg) downregulated genes linked to inflammation (*IL-1β*) and iron regulation (*hepcidin*), while higher doses (400–500 mg/kg) showed dose-dependent upregulation (*P* < 0.05). Histological studies confirmed improved intestinal villi structure and absorptive area in the 300–500 mg/kg groups. This valuable information paves the way for sustainable aquaculture practices utilizing stevioside as a functional ingredient. This study provides key insights into the multi-level responses of juvenile mullets to dietary stevioside, with potential implications for aquatic animals exposed to similar circumstances.

**Graphical Abstract:**

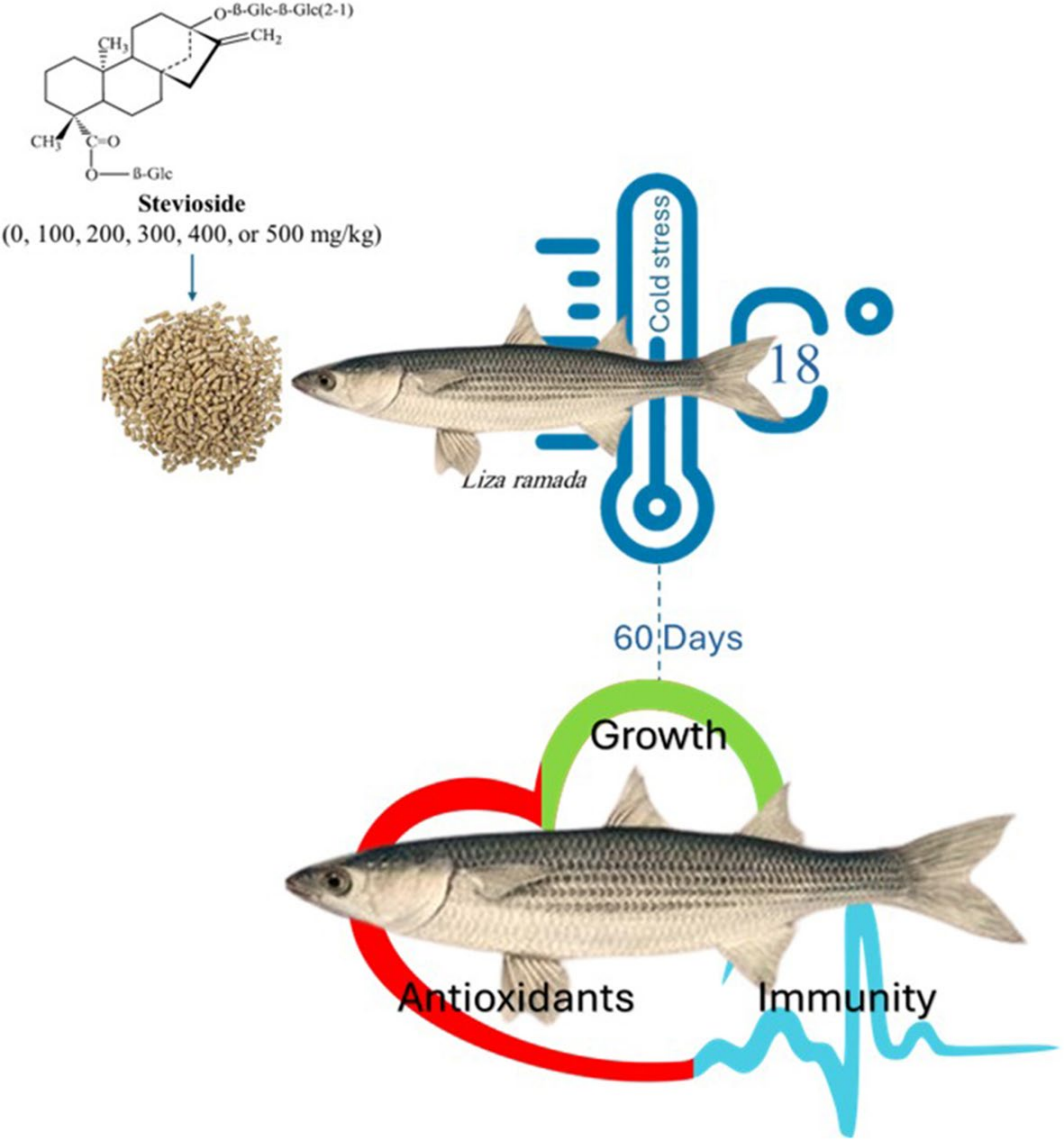

## Highlights

⌧ Stevioside enhances growth and health of *Liza ramada* juveniles under cold stress.

⌧ Stevioside boosts antioxidant activity and immunity in *L. ramada* juveniles.

⌧ Dietary stevioside promotes gut and liver health under chronic cold stress.

⌧ Stevioside modulates immune-related gene expression in *L. ramada* under cold stress.

⌧ Optimal stevioside level for mullet under long-term low temperature regime is 300-500 mg Stev/kg in the diet.

## Introduction

Aquaculture is at the forefront of global food production, driven by the increasing human population and the depletion of natural aquatic resources, necessitating sustainable and efficient farming practices that prioritize optimal fish health and growth [1, 2]. In this context, a comprehensive assessment of fish health and performance is essential and must integrate multiple complementary parameters to provide a holistic understanding of physiological and immunological status. This multidimensional approach includes evaluations of growth performance, whole-body proximate composition, serum biochemical markers, and antioxidant enzyme activities to assess production efficiency and metabolic balance. Additionally, it involves the analysis of immune responses, gene expression profiling through high-throughput molecular techniques, and histopathological examinations, particularly of the intestine, to elucidate the impacts of dietary and environmental factors on immune modulation, nutrient assimilation, and overall fish welfare [3, 4].

To optimize fish health and growth, researchers are investigating natural additives and dietary supplements as potential solutions [5–7]. In particular, various bioactive ingredients have been employed in aquaculture to enhance meat quality, bolster immune responses, and improve antioxidant capacity, contributing to overall fish welfare and product value [8, 9]. Prebiotics, non-digestible dietary components that support gut bacteria and host health, are promising [10]. Their use in fish diets has grown to improve development and feed utilization across varied species [11–14]. Plant-derived components such as polysaccharides and oligosaccharides, in particular, have demonstrated positive effects on fish health and performance [15, 16]. Their benefits are attributed to several mechanisms, including pathogen trapping, gut health enhancement, and microbiota modulation [12, 17]. Research indicates that prebiotics boost fish immune responses, including lysozyme and immunoglobulin synthesis [13, 18, 19]. Immune boost enhances fish health and resilience, contributing to more sustainable and efficient aquaculture [20]. However, further research on specific prebiotics like stevioside may uncover additional benefits for various fish species.

Stevioside, derived from *Stevia rebaudiana*, is being investigated as a prebiotic in aquaculture. It is a popular sugar replacement due to its sweetness intensity several hundred times that of sucrose and low calorie content [21, 22]. Its potential health benefits make it a promising fish prebiotic, surpassing artificial sweeteners in this regard. Stevioside may benefit gastrointestinal and systemic health due to its immunomodulatory and antioxidant properties, as demonstrated in various species and at different doses: in vitro studies on porcine intestinal epithelial cells (IPEC-J2) have shown protective effects against oxidative stress [23], while in vivo studies on broiler embryos have established safety at doses up to 5 mg per egg [24]. Furthermore, stevioside has been extensively studied in rodents and humans, showing safety even at high doses (up to 1500 mg/kg body weight/day in rats for 2 years) and extended periods of consumption [25].

In aquaculture specifically, Wang, et al. [19] found substantial improvements in liver antioxidant capacity, growth performance, and immunological function in juvenile mirror carp (*Cyprinus carpio*). Their broken-line regression analysis found that 217.68 mg/kg and 215.21 mg/kg stevioside concentrations maximized weight gain ratio (WGR) and SOD activity. Stevioside has demonstrated immunomodulatory effects in various species, including aquatic organisms, by influencing cytokine production (e.g., IL-1β, IL-8, TNF-α) and regulating immune cell functions. This enhances host defenses and reduces inflammation, suggesting its potential to improve immune function in aquaculture [26–28]. Further study is needed to understand its impact on other fish species, especially under stressful situations including poor water quality, low or high temperatures, and crowding.

Thinlip mullet (*Liza ramada*), an important aquaculture fish, thrives above 20 °C. However, its cultivation in places with seasonal temperature changes exposes it to inadequate winter temperatures [29, 30]. This cold stress significantly impacts warm-water fish, hindering their health, growth, and economic viability. Reduced feeding and metabolic rate below 18 °C lead to stunted growth, and lower productive and reproductive processes in warm-water fish [31, 32]. These negative impacts necessitate sustainable aquaculture mitigation measures in temperature-sensitive locations. Using antioxidant and immunomodulatory dietary supplements like stevioside seems promising.

Thus, this study aims to elucidate the potential modulatory effects of stevioside, administered at different dosages, on the development, biochemical composition, metabolic performance, antioxidant enzyme activity, and immunological response of juvenile *Liza ramada* subjected to chronic low-temperature stress.

## Materials and methods

### Ethical approval

This research has received ethical approval from the College of Agriculture Committee for Animal Care at Alexandria University, Egypt, with reference number AU: 19/23/07/24/3/33. All study techniques were conducted in strict adherence to the Animal Research: Reporting In Vivo Experiments (ARRIVE) guidelines v2.0 [33].

### Juvenile fish acquisition and acclimation

Juvenile thinlip mullets (Liza ramada) used in the experiment, with an initial body weight of 3.50 ± 0.07 g, were sourced from a private farm in Kafr Elsheikh City, Egypt. After acquisition, the fish were transported in a healthy state to the Baltim Research Station of the National Institute of Oceanography and Fisheries in Egypt, where the feeding trials were conducted. Upon arrival, all mullets underwent a 15-day acclimatization period. The mullet were acclimatized to 18 ºC, with this temperature chosen based on the median value of mullet occurrence [34]. During this period, they were fed the basal diet (Table 1). After acclimatization, 540 fish were randomly distributed among 18 tanks (100 L polycarbonate) and fed the experimental diets for 60 days.

**Table 1:** Feed composition (g/kg) and nutrient levels (% dry weight; n = 3) of basal diet

Feed composition^1^	(g/kg)	Nutrient levels (%)	
Fish meal (65% CP)	50	Dry matter	89.01 ± 0.35
Soybean meal (48% CP)	365	Crude protein	30.87 ± 0.11
Meat meal (55% CP)	100	Crude lipid	5.94 ± 0.15
DDGS ^2^	65	Ash	8.14 ± 0.11
Wheat bran	55	Crude fiber	7.20 ± 0.11
Rice bran	200	NFE^5^	47.84 ± 0.38
Wheat flour	40		
Sunflower oil	11		
Broken rice	75		
Mineral premix ^3^	1.5		
Vitamin premix ^4^	1.5		
Dicalcium phosphate	10		
Methionine	6		
Limestone	10		
Salt	10		
Total	1000		

^1^The ingredients are supplied by Feed Control Co., Ltd., which is located in Damro, Sidi Salem, Kafrelsheikh, Egypt

^2^DDGS = distiller's dried grains with solubles

^3^Per kilogram of premix, the mineral mixture contains: manganese (325 mg), iron (200 mg), copper (25 mg), iodine, and cobalt (5 mg)

^4^The mineral mixture includes (per kilogram of premix): vitamin A (3300 IU), vitamin D3 (410 IU), vitamin E (2660 mg), vitamin B1 (133 mg), vitamin B2 (580 mg), vitamin B6 (410 mg), vitamin B12 (50 mg), biotin (9330 mg), choline chloride (4000 mg), inositol (330 mg), para-aminobenzoic acid (9330 mg), niacin (26.60 mg), and pantothenic acid (2000 mg)

^5^*NFE* nitrogen-free extract (by difference)

### Experimental design and setup

The experiment was conducted at the research station of the National Institute of Oceanography and Fisheries in Baltim, Egypt. Employing a completely randomized design, each treatment was replicated three times. Juvenile thinlip mullets (*Liza ramada*) were stocked in tanks maintained at a constant temperature of 18.2 ± 0.21 °C, pH of 7.22 ± 0.27, dissolved oxygen concentration of 6.68 ± 0.34 mg/L, and total ammonia concentration of 0.05 ± 0.03 mg/L. Fish were reared in a controlled environment with natural light condition and 20% of the culture water was replaced daily with dechlorinated water. To manage metabolic waste, including ammonia, nitrite, and carbon dioxide and uneaten food, a siphoning system was employed. This involved using a vacuum siphon to carefully remove settled waste and uneaten food from the bottom of the tanks without disturbing the fish. Continuous aeration was provided through a compressed air pump across all experimental tanks. To ensure a constant temperature of 18 °C, each tank was outfitted with an adjustable heater and a digital thermometer for continuous monitoring.

### Stevioside source and feeding regime

Stevioside of 98.5% purity was procured from Shana Natural House in El Asafra Bahary, Alexandria, Egypt. Six dietary treatments were used in this experiment. The first group received a control diet without stevioside (Control, Stev 0). The remaining five groups received the same basal diet supplemented with increasing levels of stevioside: 100 mg/kg (Stev 100), 200 mg/kg (Stev 200), 300 mg/kg (Stev 300), 400 mg/kg (Stev 400), and 500 mg/kg (Stev 500). The specific stevioside concentrations were chosen based on a previous study by Shehata, et al. [28]. The experimental diets were formulated to include high-quality protein sources, such as Fish meal, Soybean meal, Meat meal, and distiller's dried grains with soluble (DDGS), to achieve a crude protein content of 30%. Sunflower oil provided the lipid source (5.94% crude lipid). A vitamin and mineral premix following Shehata, et al. [7] ensured a complete nutritional profile. All ingredients were carefully blended before processing into 1–2 mm pellets using a laboratory pelletizer with oils and water. The final pellets were air-dried at room temperature and stored at 4 °C. Juvenile thinlip mullets received the formulated diets (Table 1) three times daily for 60 days at a feeding rate corresponding to 3% of their body weight.

### Samples collection and measurements

Following a 60-day feeding experiment, fish in each replicate (tank) were weighed and individually counted approximately 24 h after the final feeding. The thinlip mullets were then carefully anesthetized using a 50 µl/L clove oil solution as an anticoagulant for further blood and tissue parameters analysis. Blood specimens were collected through caudal puncture into centrifuge tubes and allowed to clot at room temperature. Serum was subsequently separated by centrifugation at 3000 rpm for 10 min. Various tissues, including the liver, were collected for further analysis, which included assessments of antioxidant enzymes and gene expression. Additionally, the intestine and liver were used for histological evaluations.

#### Growth and feed utilization measurements

Following the experiment, various growth and feed utilization parameters were calculated for each treatment group [35]. These included:

$$\textrm{WG},\textrm{g}=\textrm{FW},\textrm{ g }-\textrm{IW},\textrm{ g}$$

$$\textrm{ADG},\textrm{ g}=\frac{\textrm{WG},\textrm{ g}}{\textrm{T},\textrm{ day}}$$

$$\textrm{SGR} \% /\textrm{day}=\frac{\textrm{Ln FW}-\textrm{LN IW}}{\textrm{T}} \times 100$$

$$\textrm{SR}, \%=\frac{\textrm{FN}}{\textrm{IN}}\times 100$$

$$\textrm{FI},\textrm{ g}/\textrm{fish}/\textrm{Tdays} =\frac{\textrm{Dry diet provided},\textrm{ g}-\textrm{Uneaten diet recovered},\textrm{g}}{\textrm{Fish No}.}$$

$$\textrm{FCR}=\frac{\textrm{FI},\textrm{g}}{\textrm{WG},\textrm{g}}$$

where:

WG: weight gain, g; FW: final weight, g; IW: initial weight, g; ADG: average weight gain, g; T: trial period, day; SGR: specific growth rate; SR: survival rate; FN: final number; IN: initial number; FI: feed intake, g; FCR: feed conversion ratio.

#### Chemical composition analysis

Utilizing established protocols from the Association of Official Analytical Chemists [36], the nutrient analysis of the basal diet and whole-body fish samples involved different methodologies tailored to each sample type. The moisture content of samples was evaluated by exposing them to a steady temperature of 105 °C until they reached a stable weight. To determine ash content, samples were incinerated at 550 °C for 36 h in a muffle furnace. Fat extraction, performed over the 6-h, utilized an ether extractor (SoxROC, OPSIS, Sweden). Protein content quantification followed the Kjeldahl method, where samples underwent digestion with concentrated sulfuric acid, and measurements were conducted using an automatic Kjeldahl apparatus (KD210, OPSIS, Sweden). Fiber content in diet samples was determined by following the procedure outlined by Van Soest, et al. [37].

#### Biochemical parameters analysis

Five fish per replicate were allocated for serum collection. The biochemical parameter in serum was examined using a biochemical kit obtained from Bio-Diagnostic Co. in Cairo, Egypt. The measured biochemical parameters are Glucose (Glu, mmol/L, Cat. No. GL 13 20), Total protein (TP, g/dL, Cat. No. TP 20 20), Albumin (Alb, g/dL, Cat. No. AB 10 10), Globulin (Glob, by differences), Total cholesterol (T-CHOL, mg/dL, Cat. No. TC 20 10), Triglyceride (TG, mg/dL, Cat. No. TG 20 11), Alanine aminotransferase (ALT) and Aspartate aminotransferase (AST) (U/L, Cat. Nos. AT 10 34 and AT 10 45), Urea (mg/dL, Cat. No. UR 21 10), and Creatinine (mg/dL, Cat. No. CR 12 50). Adhering to the instructions outlined in the respective kit packages, the analysis of biochemical blood profiles was conducted to ensure the accuracy and consistency of serum parameter assessments. The Model CBC Micros ABX, France automatic analyzer for clinical chemistry and hematology assays was used, along with Diatron Q.C Reagents strips [P500 kinetic & Quality control].

#### Assessment of antioxidant enzyme activities

Livers from three fish per tank (9 fish/treatment) were carefully excised, immediately placed on ice, and then stored at −20 °C for antioxidative enzyme analysis. Liver samples were homogenized in cold 0.86% NaCl solution using a VEVOR FSH-2 A homogenizer according to the manufacturer's instructions. The homogenate was centrifuged at 12,000 rpm for 10 min at 4 °C. The resulting supernatant was used for both protein content determination and antioxidative enzyme activity assays. Total protein content was determined using the Bradford method [38]. Briefly, the supernatant was diluted appropriately, and 10 μL of diluted sample or bovine serum albumin (BSA) standards (0–2000 μg/mL) were added to 200 μL of Bradford reagent in a 96-well microplate. After 5 min of incubation at room temperature, the absorbance was measured at 595 nm using a microplate spectrophotometer. Protein concentrations were calculated using a BSA standard curve. Antioxidative enzyme activities were measured using colorimetric methods. Superoxide dismutase (SOD) activity was measured by the inhibition rate of autocatalytic adrenochrome synthesis at 550 nm, following Misra and Fridovich [39]. Catalase (CAT) activity was assessed by tracking the degradation of hydrogen peroxide at 280 nm, according to Góth [40]. Glutathione peroxidase (GPx) activity was determined by monitoring the oxidation of NADPH with absorbance readings at 412 nm, following Arun, et al. [41]. All of these studies were carried out using a microplate spectrophotometer, ensuring accurate and repeatable detection of enzyme activity.

#### Evaluation of immune response

We assessed the serum activities using a 96-well microplate turbidimetric assay, following the method described by Lygren, et al. [42]. The test used lyophilized *Micrococcus lysodeikticus* cells (Sigma-Aldrich, India) as the substrate. We inserted 10 µl of serum samples into 96 microplate tubes throughout this process. The substrate combination of 190 l of 0.2 mg/ml *M. lysodeikticus* in PSB (pH = 7.4) was then added. We measured changes in cloudiness at 450 nm after 1 and 5 min of gently stirring the solution at room temperature. We assessed enzyme activity by calculating the quantity of enzyme required to generate a 0.001 unit per minute drop in absorbance.

According to Wang, et al. [43], the bactericidal activities of serum were measured spectrophotometrically at 570 nm using modified techniques based on Gallage, et al. [44]. In short, using a micro-tube rotator (Wavex–Tube Rotator E11270), serum samples were combined with a bacterial culture (*Streptococcus agalactiae*, 1.4 × 108) in a 1:1 ratio (50 μl sample: 50 μl bacterial suspension) and incubated at 25 °C for 2.5 h. After incubating for 15 min at 25 °C with moderate shaking, we transferred the mixtures to 96-microplate tubes and mixed them with 15 μl of 3-(4,5-Dimethyl-2-thiazolyl)−2,5-diphenyl-2H-tetrazolium bromide (MTT) (Sigma-Aldrich, Egypt) solution (5 mg/ml). Dissolving the formazan required fifty microliters of dimethyl sulfoxide (DMSO). We used a bacterial suspension in PBS as a positive control, without serum samples. We made three measurements of the optical density (OD570) and expressed the antibacterial activities as a percentage of the growth of *S. agalactiae* being inhibited relative to the positive control.

$$S. agalactiae \;\textrm{inhibition}\; \%=\frac{{\textrm{OD}}_{\textrm{Control}}-{\textrm{OD}}_{\textrm{Sample}}}{{\textrm{OD}}_{\textrm{Control}}}\times 100$$

The respiratory burst activities in whole blood were determined using a nitroblue tetrazolium (NBT) assay, following the modified protocol by Secombes [45] at 630 nm. Furthermore, we assessed the activities of the alternative complement pathway (ACP) using serum samples, following the methodologies described by El Basuini, et al. [31].

#### Gene expression analysis

The fish liver samples (5 fish/tank) were isolated and thereafter stored at a temperature of −80 °C for analysis. The liver samples were partitioned into pieces weighing 500 mg each, then transferred to mortar and homogenized with lysis solution (Geneaid GenozolTri RNA Kit, Korea). Subsequently, the mixture was centrifuged to obtain the aqueous layer for separating the RNA by column according to manufacturer (Geneaid, Korea). After isolating RNA, The RNA yield integrity, quantities, and quality were checked by NanoDrop spectrophotometer (BioDrop, England). The normalization of RNA sample concentration was performed to be 50 ng for each sample.

We used the one-step RT q-PCR Syber Green Kit (enzymnomics, Korea) for real-time PCR analysis, following the primer recommendations in Table 2. The reactions were performed according to the manufacturer's procedure for expression analysis. The reaction conditions were as follows: We held the reaction at 50 ℃ for 30 min (for cDNA synthesis), then started the PCR reaction at 95 ℃ for 10 min, then shifted to 95 ℃ for 5 s, and finally annealed at 60 ℃ for 30 s using different primers for all the targeted genes. We repeated this process for 45 cycles. We conducted the amplification using the CFX-3110 2-step real-time PCR system (BIORAD, USA). Melting curves proved the specificity of real-time PCR amplification. This ensured the amplification of only one PCR product at the target size. We conducted the sample quantification in triplicate for each treatment. We performed expression analysis using the 2^−∆∆Ct^ method [46]; where the fold change (2^−ΔΔCt^) = 1 (control); < 1 = downregulated; > 1 = upregulated. The expression of the studied genes was normalized using the housekeeping gene *β*-actin gene expression.

**Table 2:** RT-qPCR primer sequences used in this study

Gene	Sequences of forward and reverse primers (5’−3’)	Annealing Temperature (^ο^C)	Amplicaon size (bp)	Accession numbers
*Hep*-F	ATGAAGGCATTCAGCATTGC	60	221	MH674371.1
*Hep*-R	TCAGAACTTGCAGCAGAAGC			
*il1β*-F	GAGGAGCTTGGTGCAGAACA		190	OY741297.1
*il1β*-R	CTTTGTTCGTCACCTCCTCCA			
*β- actin*-F	CCACGAGACCACCTACAACA		270	OY741309.1
*β- actin*-R	CTCTGGTGGGGCAATGAT			

*Hep Hepcidin*, *il1β Interleukin 1-β*

#### Histological observation

The histological examination was conducted at the completion of the experiment. The intestine and liver specimens were promptly immersed in a 10% neutral buffered formalin solution for a duration of 48 h. Following fixation, the tissue specimens underwent processing. The tissue samples underwent dehydration using a series of increasing concentrations of ethyl alcohol. They were then treated with xylene to remove any remaining impurities. Next, the samples were embedded in paraffin wax and cut into several slices that were 5 μm thick using a rotary microtome (RM 20352035; Leica Microsystems, Wentzler, Germany). Finally, the sections were placed onto clean slides. The paraffin slices underwent rehydration and were stained with Hematoxylin and Eosin (H and E) using the methods outlined in Bancroft and Gamble [47] for overall histomorphology. Subsequently, a digital camera (Leica EC3, Leica, Germany) coupled with a microscope (Leica DM500) was used to record multiple typical photomicrographs from the stained sections. The morphometric analysis used three slides per treatment (one per replicate). Each slide was checked for three fields of view. The morphometric analysis was confirmed through two separate individuals. The morphometric analysis employed an automated image analysis system (ImageJ; Bethesda, MD, USA) to evaluate villus height (μm; from the tip to the base of the villus), villus width (μm; at the midheight of villi), and muscularis thickness, following the methodology described by Schneider, et al. [48]. Measurements were taken in micrometers (μm) and the collected data were analyzed statistically.

### Statistical Analysis

We used SPSS Software V26 to calculate the mean and standard error of the mean (SEM). One-way analysis of variance (ANOVA) followed by Tukey's post hoc test was applied to analyze the data. The Shapiro–Wilk test was used to assess normality, and the equality of variance was confirmed using Levene’s test. A *p*-value threshold of 0.05 was employed to determine statistical significance.

## Results

### Growth performance, feed utilization, and survival rate

Table 3 presents the effects of varying dietary stevioside levels (Stev) on the growth variables, feed efficacy, and survival of juvenile thinlip mullet (*L. ramada*) under chronic low-temperature stress conditions. Supplementation with higher Stev doses (300–500 mg/kg diet) significantly improved FBW, WG, ADG, SGR, and FI compared to the control and lower Stev doses (100–200 mg/kg). Notably, the 400 and 500 mg/kg Stev groups exhibited the highest FBW, WG, ADG, and SGR values. Feed conversion ratio (FCR) was also lowest in the 400 and 500 mg/kg Stev groups. Survival (SR) remained unaffected across treatments.

**Table 3:** Impact of stevioside on Thinlip mullet (*Liza ramada*; IBW = 3.50 ± 0.07 g) growth and survival under chronic low temperature stress

Parameters	Dietary stevioside levels (mg/kg diet)					
	Cont _0 mg/kg_	Stev _100 mg/kg_	Stev _200 mg/kg_	Stev _300 mg/kg_	Stev _400 mg/kg_	Stev _500 mg/kg_
FBW (g)	9.50 ± 0.29^b^	9.93 ± 0.07^b^	9.87 ± 0.13^b^	10.40 ± 0.10^a^	10.77 ± 0.07^a^	10.47 ± 0.03^a^
WG (g)	6.00 ± 0.25^b^	6.43 ± 0.05^b^	6.37 ± 0.11^b^	6.90 ± 0.09^a^	7.27 ± 0.06^a^	6.97 ± 0.04^a^
ADG (g/fish/d)	0.10 ± 0.01^c^	0.11 ± 0.01^ab^	0.12 ± 0.01^bc^	0.12 ± 0.01^a^	0.12 ± 0.01^a^	0.12 ± 0.02^a^
SGR (%/d)	1.66 ± 0.05^c^	1.74 ± 0.01^bc^	1.73 ± 0.02^c^	1.81 ± 0.02^ab^	1.87 ± 0.01^a^	1.83 ± 0.03^a^
SR (%)	100	100	100	100	100	100
FI (g/fish)	9.90 ± 0.17^b^	10.16 ± 0.04^b^	10.12 ± 0.08^b^	10.44 ± 0.06^a^	10.66 ± 0.04^a^	10.48 ± 0.02^a^
FCR	1.66 ± 0.05^a^	1.58 ± 0.01^abc^	1.59 ± 0.02^ab^	1.51 ± 0.01^bcd^	1.47 ± 0.01^d^	1.50 ± 0.03^ cd^

*IBW* initial body weight; *FBW* final body weight; *WG* weight gain; *ADG* average daily gain; *SGR* specific growth rate; *SR* survival rate; *FI* feed intake; *FCR* feed conversion ratio

Values represent means ± SE (n = 3), and values within the same row with different letters were significantly different (*p* ≤ 0.05, one-way ANOVA)

### Body chemical composition

The proximate composition analysis of whole-body juvenile thinlip mullet (*L. ramada*) following the 60-day feeding trial under cold stress conditions revealed no significant differences (*p* > 0.05) in moisture, protein, lipid, and ash content across the experimental groups fed varying dietary stevioside levels (0–500 mg/kg diet) (Table 4).

**Table 4:** Proximate profiles of the whole-body of thinlip mullet juveniles fed the experimental diets for 60 days under cold stress

Parameters	Dietary stevioside levels (mg/kg diet)					
	Cont _0 mg/kg_	Stev _100 mg/kg_	Stev _200 mg/kg_	Stev _300 mg/kg_	Stev _400 mg/kg_	Stev _500 mg/kg_
Moisture (%)	72.46 ± 0.42	72.29 ± 0.20	72.52 ± 0.16	72.63 ± 0.28	72.62 ± 0.32	72.12 ± 0.26
Protein (%)	19.33 ± 0.37	19.43 ± 0.47	19.44 ± 0.18	19.24 ± 0.04	19.23 ± 0.07	19.68 ± 0.14
Lipid (%)	6.02 ± 0.12	6.11 ± 0.02	6.11 ± 0.08	5.94 ± 0.16	6.00 ± 0.11	6.04 ± 0.13
Ash (%)	2.19 ± 0.09	2.16 ± 0.37	2.22 ± 0.07	2.22 ± 0.10	2.16 ± 0.17	2.17 ± 0.25

Values represent means ± SE (*n* = 3), and values within the same row with different letters were significantly different (*p* ≤ 0.05, one-way ANOVA)

### Biochemical Parameters

Table 5 reveals the significant impacts of dietary stevioside supplementation on the serum biochemical analysis of *L. ramada* following the 60-day cold stress feeding trial. The control group exhibited higher serum glucose and total cholesterol levels compared to fish-fed stevioside-supplemented diets. Serum glucose concentrations decreased significantly with stevioside inclusion, regardless of dose level. Total cholesterol levels gradually declined as dietary stevioside increased from 100 to 500 mg/kg. In contrast, total protein and globulin levels were elevated in the serum of fish receiving stevioside, with the highest total protein observed at doses over 100 mg/kg and peak globulin at 300 mg/kg. However, serum albumin, triglycerides, ALT, AST, urea, and creatinine remained unaffected (*p* > 0.05) across all dietary treatments.

**Table 5:** Impact of stevioside supplementation on serum biochemistry of *Liza ramada* exposed to chronic low temperature stress

Parameters		Dietary stevioside levels (mg/kg diet)				
	Cont _0 mg/kg_	Stev _100 mg/kg_	Stev _200 mg/kg_	Stev _300 mg/kg_	Stev _400 mg/kg_	Stev _500 mg/kg_
GLU (mmol/L)	9.22 ± 0.19^a^	6.13 ± 0.10^b^	6.10 ± 0.02^b^	6.09 ± 0.06^b^	6.08 ± 0.07^b^	5.93 ± 0.09^b^
TP (g/dl)	4.21 ± 0.08^c^	4.80 ± 0.09^b^	5.33 ± 0.16^a^	5.42 ± 0.10^a^	5.45 ± 0.11^a^	5.44 ± 0.07^a^
ALB (g/dl)	2.26 ± 0.38	2.38 ± 0.49	2.40 ± 0.49	2.34 ± 0.42	2.85 ± 0.38	3.08 ± 0.11
GLOB (g/dl)	1.95 ± 0.31^b^	2.42 ± 0.0.40^ab^	2.93 ± 0.33^ab^	3.08 ± 0.41^a^	2.60 ± 0.31^ab^	2.37 ± 0.04^ab^
T-CHOL (mg/dl)	19.33 ± 0.88^a^	16.00 ± 0.58^bc^	16.33 ± 0.88^b^	16.00 ± 0.58^bc^	14.00 ± 0.58^ cd^	13.67 ± 0.33^d^
TG (mmol/L)	63.00 ± 1.73	63.33 ± 2.18	64.00 ± 1.53	64.67 ± 0.66	65.33 ± 2.40	65.00 ± 2.65
ALT (U/L)	22.07 ± 1.08	21.71 ± 1.67	20.29 ± 1.55	21.49 ± 0.82	20.28 ± 1.43	21.87 ± 1.30
AST (U/L)	34.62 ± 1.73	35.53 ± 1.66	33.25 ± 1.74	33.19 ± 1.13	33.78 ± 1.44	32.44 ± 2.08
Urea (mg/dl)	25.67 ± 0.88	25.33 ± 1.45	23.00 ± 0.99	24.33 ± 0.88	23.33 ± 0.88	23.00 ± 1.53
Creatinine (mg/dl)	0.51 ± 0.11	0.49 ± 0.07	0.48 ± 0.10	0.49 ± 0.04	0.48 ± 0.08	0.46 ± 0.06

GLU: glucose; TP: total protein; ALB: albumin; GLOB: globulin; T-CHOL: total cholesterol; TG: triglyceride; ALT: alanine aminotransferase; AST: aspartate aminotransferase. Values represent means ± SE (n = 3), and values within the same row with different letters were significantly different (*p* ≤ 0.05, one-way ANOVA)

### Activities of antioxidant enzymes

**Fig. 1:**
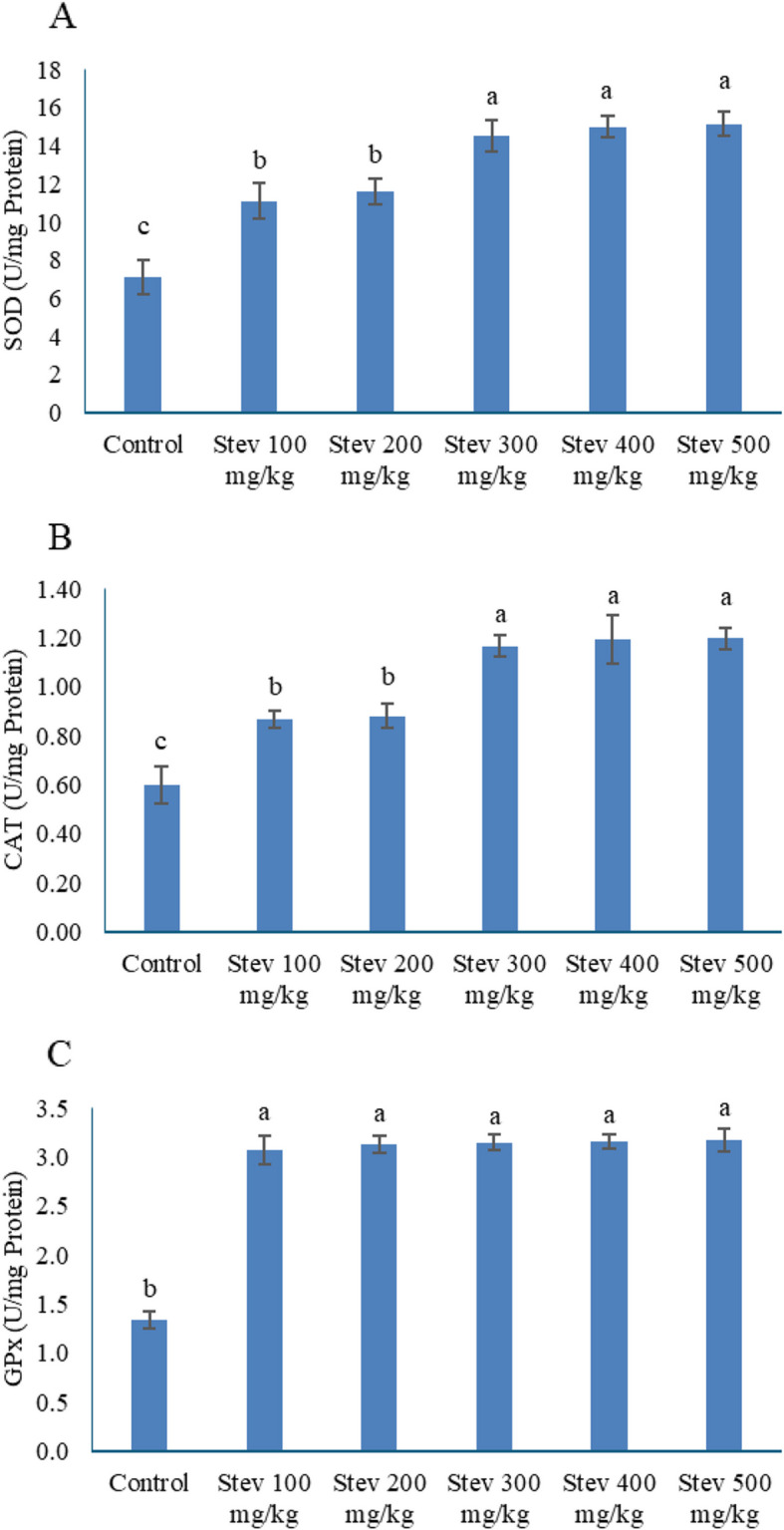
Liver antioxidant parameters in *Liza ramada* after 60 days of feeding trial on stevioside levels. **A** SOD: Superoxide dismutase; **B** CAT: Catalase; **C** GPx: Glutathione peroxidase. Values represent means ± SE (n = 3), and bars with different letters were significantly different (*p* ≤ 0.05, one-way ANOVA)

The antioxidant status in the liver of *L. ramada* after the 60-day feeding trial revealed that dietary stevioside supplementation significantly enhanced superoxide dismutase (SOD), catalase (CAT), and glutathione peroxidase (GPx) activities compared to the control group (Fig. 1). Fish fed diets with higher stevioside levels (≥ 300 mg/kg) exhibited greater SOD and CAT activities than lower doses. However, GPx activity did not significantly differ across the various stevioside concentrations.

### Immune system responses

The 60-day cold stress feeding trial showed that dietary stevioside supplementation significantly boosted the innate immune response in *L. ramada* (Table 6). Fish fed stevioside-supplemented diets exhibited higher lysozyme activity, bactericidal activity, nitro-blue tetrazolium (NBT) reduction ability, and alternative complement pathway (ACH50) activity compared to the control group. While bactericidal activity, NBT, and ACH50 did not differ substantially across stevioside doses, lysozyme activity peaked at doses over 400 mg/kg stevioside.

**Table 6:** Immune system responses of *Liza ramada* fed diets supplemented with different levels of stevioside under cold stress for 60 days

Parameters		Dietary stevioside levels (mg/kg diet)				
	Cont _0 mg/kg_	Stev _100 mg/kg_	Stev _200 mg/kg_	Stev _300 mg/kg_	Stev _400 mg/kg_	Stev _500 mg/kg_
Lysozyme activity (U/ml)	184.28 ± 3.21^c^	297.71 ± 6.86^b^	300.30 ± 1.26^b^	324.60 ± 3.02^a^	328.50 ± 5.43^a^	333.06 ± 5.35^a^
Bactericidal activity %	6.60 ± 0.18^b^	10.22 ± 0.65^a^	10.41 ± 0.53^a^	10.39 ± 0.32^a^	10.43 ± 0.37^a^	10.61 ± 0.64^a^
NBT %	0.17 ± 0.01^b^	0.26 ± 0.01^a^	0.27 ± 0.01^a^	0.28 ± 0.02^a^	0.28 ± 0.01^a^	0.29 ± 0.01^a^
ACH50 (U/ml)	37.68 ± 1.45^b^	53.32 ± 1.81^a^	53.61 ± 1.79^a^	55.88 ± 2.20^a^	56.02 ± 2.38^a^	56.87 ± 1.78^a^

*NBT* Nitro-blue Tetrazolium; *ACH50* Serum alternative complement pathway

Values represent means ± SE (n = 3), and values within the same row with different letters were significantly different (*p* ≤ 0.05, one-way ANOVA)

### The expression of *IL-1β* and *hepcidin* mRNA

**Fig. 2:**
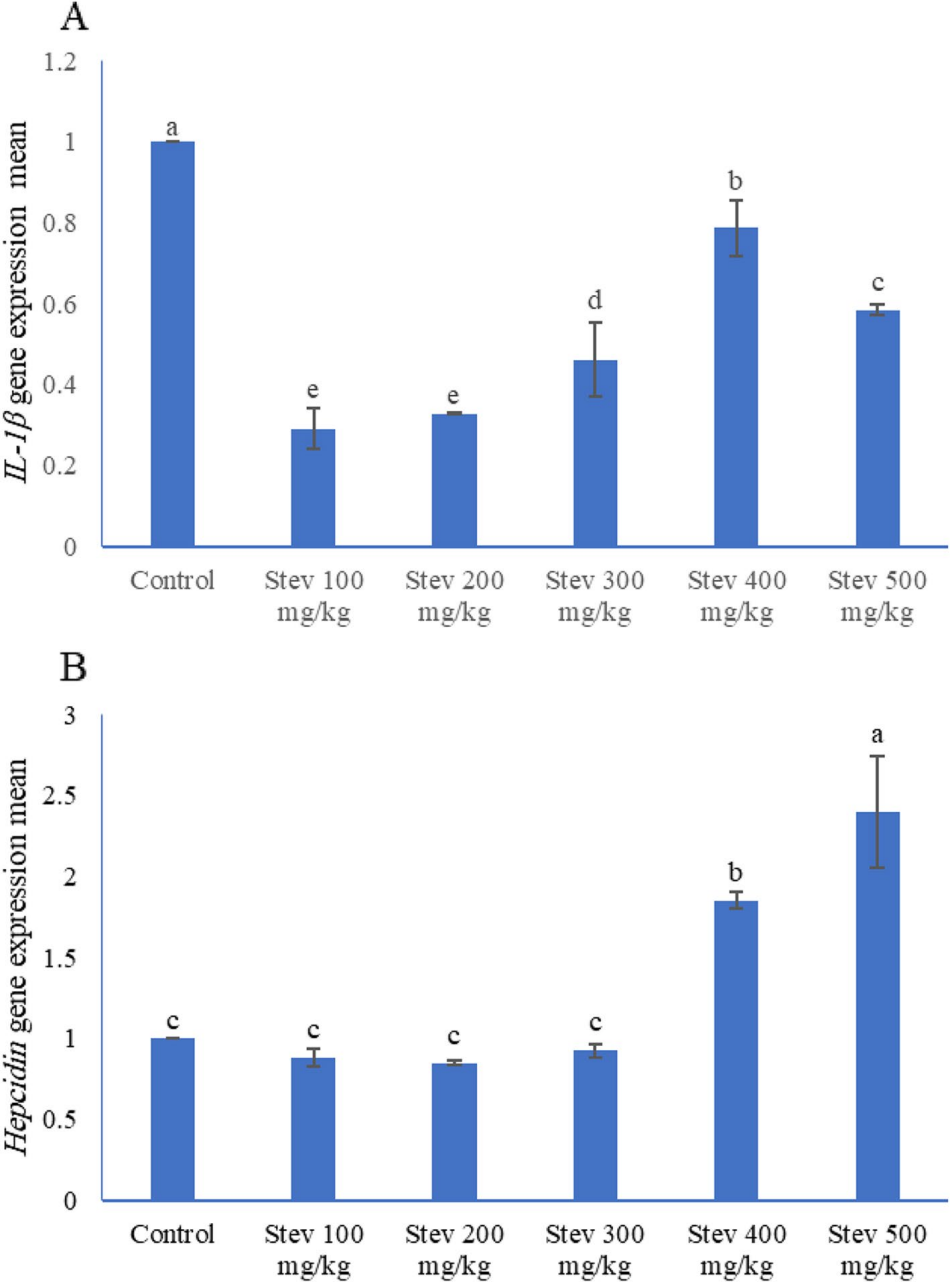
Gene expression of (**A**) *IL-1β*: *interleukin-1 beta* and (**B**) *Hepcidin* genes on stevioside levels in Thinlip Mullet (*Liza ramada*) juveniles after a 60-day feeding trial. *IL-1β*, Interleukin-1β. Values represent means ± SE (n = 3), and bars with different letters were significantly different (*p* ≤ 0.05, one-way ANOVA)

Figure 2 shows the gene expression profiles of *interleukin-1β* (*IL-1β*) and *hepcidin* in *L. ramada* fish following a 60-day feeding trial under chronic cold stress. The gene expression analysis of *IL-1β* and *hepcidin* revealed a dose-dependent modulation by dietary stevioside supplementation. Lower stevioside doses (100–300 mg/kg) resulted in downregulation of both *IL-1β* and *hepcidin* genes compared to higher stevioside doses (≥ 400 mg/kg). The control group exhibited higher *IL-1β* expression compared to treatment groups receiving stevioside at 100–400 mg/kg. Conversely, hepcidin expression was gradually lowest at doses of 200 mg/kg, 100 mg/kg, 300 mg/kg, control, and 400 mg/kg, then peaked at stevioside doses up to 500 mg/kg.

### Histological observation

**Fig. 3:**
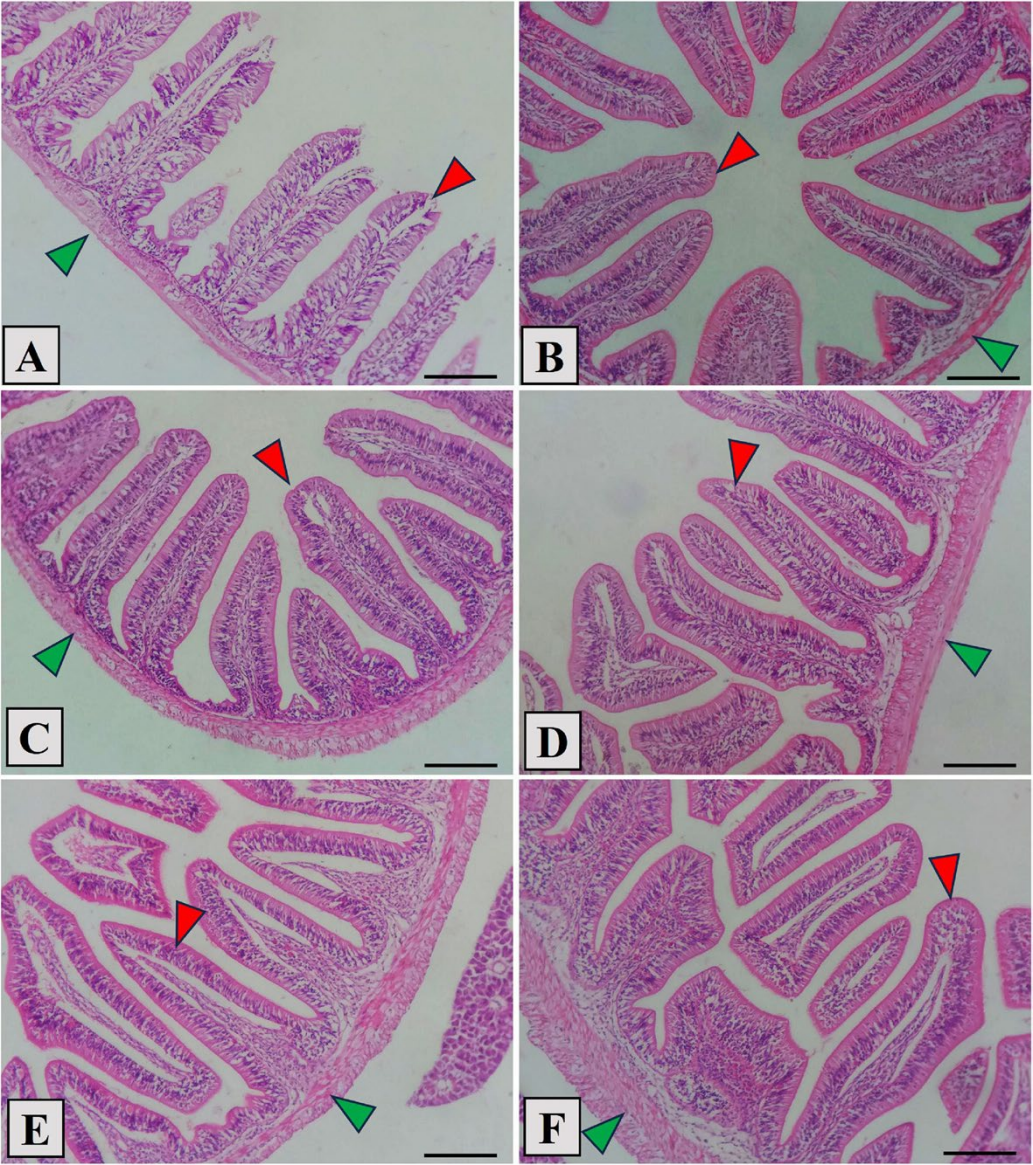
Photomicrograph showing the histological structure of middle segment of *Liza ramada* juveniles’ intestine in the control group (**A**), as well as stevioside-treated groups at ascending levels (**B**; 100, **C**; 200, **D**; 300, **E**; 400, **F**; 500 mg/kg). The control group displayed short intestinal villi (red arrowhead), vacuolated enterocytes. The intestinal wall (green arrowhead) and villi exposed evident normal structure with improved histomorphology by increased levels of stevioside. Stain H and E. Bar = 100 µm

The *L. ramada* intestine's histopathological structure showed that the intestinal wall and intestinal villi were intact in all the groups that were studied (Fig. 3A–F). The control group's intestine displayed short intestinal villi with slightly vacuolated enterocytes, somewhat separated lamina propria sub mucosa and thin intestinal wall. In the stevioside-supplemented groups, the histopathology investigation showed improved intestinal mucosa with well-arranged enterocytes. Moreover, the morphometric analysis displayed significant increase in villous height, villous width, and muscularis thickness with increasing stevioside dose (Fig. 3B–F and Table 7), especially at middle and high amounts of stevioside (300, 400, and 500 mg/kg, respectively) (Fig. 3D–F).

**Table 7:** Intestinal morphometry indices of *Liza ramada* fed diets supplemented with different levels of stevioside under cold stress for 60 days

Parameters		Dietary stevioside levels (mg/kg diet)				
	Cont _0 mg/kg_	Stev _100 mg/kg_	Stev _200 mg/kg_	Stev _300 mg/kg_	Stev _400 mg/kg_	Stev _500 mg/kg_
Villus height (μm)	135.53 ± 8.52^e^	258.73 ± 14.32^d^	287.59 ± 8.69^d^	370.41 ± 15.62^c^	429.97 ± 9.94^b^	473.04 ± 8.31^a^
Villus width (μm)	72.01 ± 5.34^c^	77.09 ± 1.62^bc^	80.70 ± 2.58^bc^	89.33 ± 4.50^b^	104.41 ± 7.96^a^	116.28 ± 3.82^a^
Muscularis thickness (μm)	32.65 ± 1.93^d^	29.64 ± 2.07^d^	42.07 ± 1.37^c^	53.92 ± 3.71^b^	52.93 ± 1.16^b^	68.93 ± 1.47^a^

Values represent means ± SE (*n* = 3), and values within the same row with different letters were significantly different (*p* ≤ 0.05, one-way ANOVA)

**Fig. 4:**
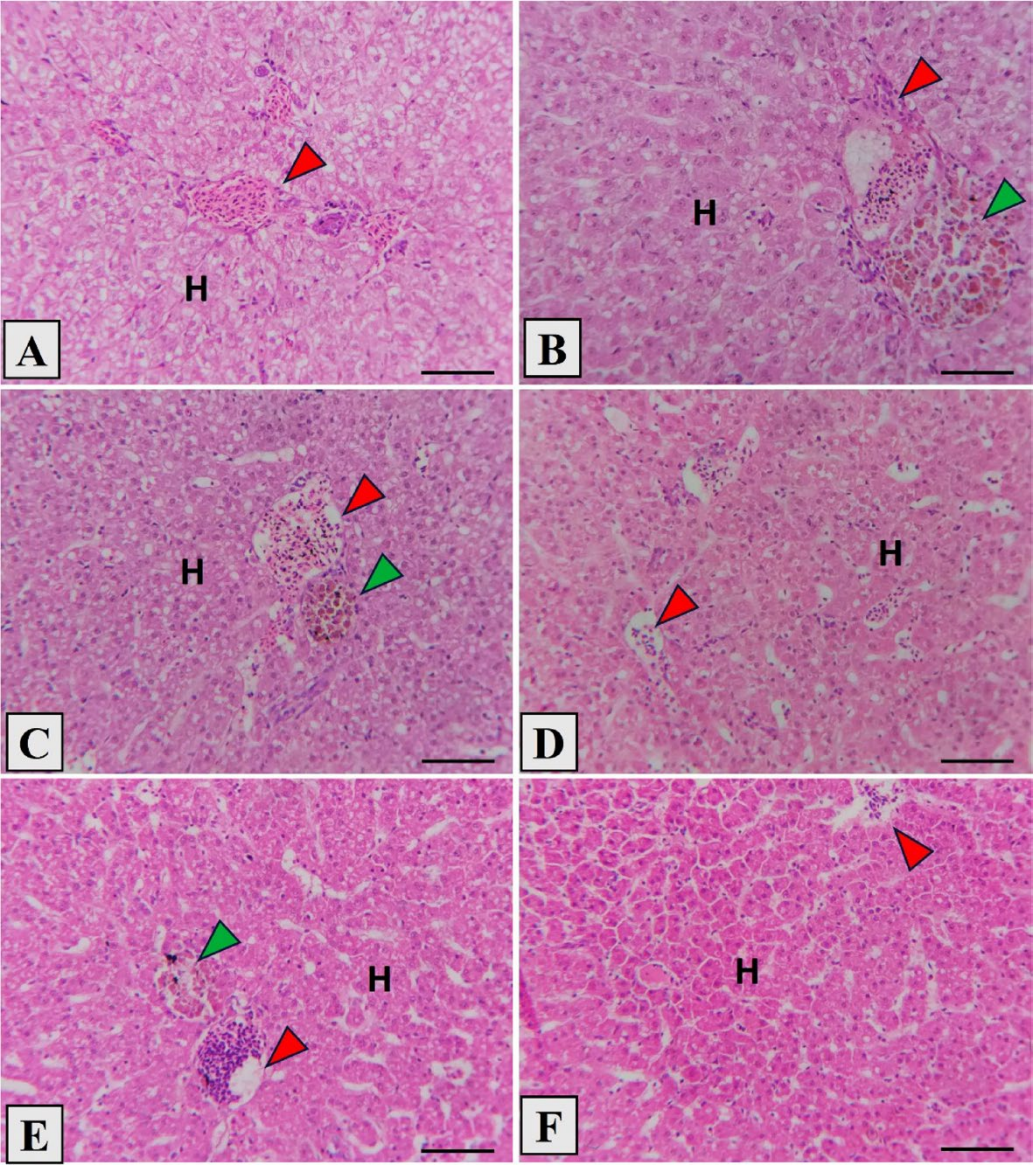
Photomicrograph showing the histological structure of *Liza ramada* juveniles’ liver in the control group (**A**), as well as stevioside-treated groups at ascending levels (**B**; 100, **C**; 200, **D**; 300, **E**; 400, **F**; 500 mg/kg). The liver presented normal appearance of hepatocytes (**H**) arranged in hepatic cords around hepatic central vein (red arrowhead), Melanomacrophage (green arrowhead) with slight vacuolation of hepatocytes in **A**-**D**. The high levels of stevioside (E, F) revealed normal hepatic architecture with leukocytic aggregation and increased glycogen deposition. Stain H and E. Bar = 50 µm

The histopathological examination of the liver in all experimental groups revealed a normal spongy appearance of hepatic parenchyma; polyhedral hepatocytes with vesicular nuclei arranged in hepatic cords around the hepatic central vein (Fig. 4A-F) with slight vacuolation in the control, low, and moderate levels of stevioside (Fig. 4A-D). The groups supplemented by high levels (400, 500 µg/kg) of stevioside revealed an enhanced appearance of hepatic architecture with leukocytic aggregation (Fig. 4E) and increased glycogen deposition (*P* < 0.05) (Fig. 4F).

## Discussion

The sustainable advancement of aquaculture relies on enhancing species'biological traits, management practices, aquatic environment, and feed regimes [49, 50]. To lay the groundwork, the use of feed additives plays a crucial role in improving fish health, promoting growth, enhancing antioxidant defence, and boosting immunity [51, 52]. Feed modifications, such as incorporating stevioside from *Stevia rebaudiana*, have shown potential in improving fish performance and immune responses [53]. This study investigates the effects of stevioside on the physiological and immunological responses of juvenile thinlip mullet (*L. ramada*) under chronic low-temperature stress.

Despite the growing popularity of *L. ramada* cultivation, critical knowledge required for its successful aquaculture remains limited. In aquaculture, growth performance parameters serve as essential metrics for evaluating production efficiency [7, 54]. These parameters are influenced by a complex interplay of environmental factors, genetic predisposition, and dietary quality and quantity. Consequently, they provide a valuable tool for assessing the effectiveness of various diets and supplements in optimizing fish farming practices [55, 56]. The present study demonstrates that dietary supplementation with Stev enhances growth and feed utilization of thinlip mullet. These findings align with the observations on *Cyprinus carpio*, where Stev supplementation similarly promoted growth performance [19].

Previous research has demonstrated that stevioside can enhance food palatability, leading to improved growth performance in piglets [57]. Research conducted on common carp showed that growth indices were greatly improved by a stevia extract concentration of 2000 mg/kg [58]. The attraction activity for aquatic animals tested is parallel with the concentrations of stevioside [59]. Nevertheless, Stev supplementation had no significant effect on tilapia growth, feed intake, or proximate composition [60]. Animal species variations in attraction activity may explain the observed discrepancies [19, 61–63]. Different pathways claim that the inclusion of Stev in *L. ramada* diets promotes growth. These factors result in increased food palatability, which leads to a higher average daily amount of feed consumed [64], attractive properties influencing aquatic animals [59], different animals'digestive enzymes can't break down Stev into Steviol [65], and they change the gut microbiota in ways that help good bacteria grow and make digesting enzymes work better [66, 67], safeguarding the stomach with bioactive compounds for improved nutrient assimilation [68, 69], bactericidal traits in acidic conditions, and the dampening of the pro-inflammatory response [70–72]. Furthermore, researchers hypothesize that stevioside promotes an enhanced immune response and growth [73].

The whole-body nutritional formation of juvenile thinlip mullet (*L. ramada*) fed diets supplemented with varying amounts of stevioside for 60 days was assessed. Our findings show a negligible improvement in the whole-body analysis, which revealed consistent values for moisture, protein, fat, and ash content across all treatments. Additionally, previous research indicated that stevioside had no significant effect on the moisture, protein, fat, or ash content in mirror carp [19].

Serum biochemical indicators serve as reflections of the physiological and metabolic condition of fish [74]. It is also an indicator of liver health and toxicological status [75]. The serum biochemical profiles of *L. ramada* were determined after a feeding study lasting sixty days. When compared to the control diet, the treatment groups showed noticeably lower blood glucose and cholesterol values. Across the different stevioside concentrations, blood glucose levels showed no variations when stevioside supplementation was implemented. Furthermore, there was a steady decrease in blood cholesterol levels as stevioside concentrations rose. Furthermore, diet-fed groups that received stevioside at doses greater than 100 mg/kg showed the highest levels of total protein in their blood serum due to stevioside supplementation. It's interesting to note that across all experimental diets, blood concentrations of globulin, albumin, triglycerides, ALT, AST, urea, and creatinine did not change. Consistent with our results, recent studies showed that the addition of Stev did not affect mirror carp blood glucose levels, which were similar to those of the control group [60]. The elevation of serum ALT and AST levels is often indicative of liver injury, as these transaminases are released into circulation when hepatocytes are damaged [76]. Wang et al. found that varying amounts of Stev had no impact on the activity of serum alanine aminotransferase (ALT), aspartate aminotransferase (AST), triglycerides (TG), acid phosphatase (ACP), and alkaline phosphatase (ALP) [19].

SOD, CAT, and GPx are key antioxidant enzymes that play critical roles in mitigating oxidative stress in biological systems [54, 77]. SOD is responsible for dismutating superoxide radicals into hydrogen peroxide and oxygen [39], while CAT efficiently decomposes hydrogen peroxide into water and oxygen [78]. GPx, on the other hand, protects cells from oxidative damage by reducing lipid hydroperoxides and hydrogen peroxide, using glutathione as a substrate [79]. These enzymes collectively form a robust defense mechanism against reactive oxygen species, helping to maintain cellular redox balance and protect organisms from oxidative damage. Supplementing with stevioside significantly increased the levels of SOD, CAT, and GPx activity compared to the control group, when assessing the activity of antioxidant enzymes. Fish groups fed a diet richer in stevioside (≥ 300 mg/kg) exhibited higher levels of catalase and SOD activity compared to the other groups. Notably, there were no appreciable variations in GPx activity across the different stevioside concentrations. The findings of a recent experiment demonstrated that Stev might boost the activity of SOD in the fish liver, but it had no effect on CAT activity or GPx concentration. Thus, the influence of Stev on antioxidants might be one of the reasons for hepatoprotective effects [80]. The findings from a prior study revealed that the addition of Stev increased the activity of SOD in the liver of *Cyprinus carpio* (common carp), while it did not affect CAT activity or MDA content [19], indicating a nuanced and selective impact of Stev on specific components of the antioxidant defense system in this species.

The impact of stevioside supplementation on the immunological response of *L. ramada* during a 60-day feeding study. Serum lysozyme activity holds significance as a key element in the innate immune response against microbial pathogens [54]. Lysozymes play a vital role in restraining bacterial proliferation and colonization by actively targeting cell wall polysaccharides. This action results in the breakdown of the bacterial cell wall, ultimately leading to the demise of the microorganisms [71, 81].

The groups that received stevioside supplementation showed substantially elevated levels of lysozyme, bactericidal, NBT%, and ACH50 activity compared to the control group. Supplementation of mullet diets with stevioside yielded comparable impacts on the immune system [28].

The field of gene expression studies has evolved from the traditional reductionist approach of single-gene sequencing to advanced high-throughput techniques, including omics technologies such as nutrigenomics in aquaculture. This progressive shift in methodology has not only deepened our comprehension of biological markers associated with nutrition-related diseases but has also bolstered our capacity to recommend judicious feed additives for maintaining a stable immune status in aquatic animals [82]. Research has shown that down-regulated cytokines in fish are associated with pro-inflammatory responses, whereas up-regulated anti-inflammatory cytokines can reduce immune inflammatory reactions [83]. Specifically, *IL-1β*, a pro-inflammatory cytokine, exerts influence over eicosanoid production, phagocyte function, lymphocyte proliferation, and stimulation through MHC-presented antigens [84]. Serving as a crucial mediator in inflammation, *IL-1β* plays a pivotal role. Additionally, *hepcidin*, responsible for iron regulation [85], functions as an indispensable antimicrobial peptide, playing a critical role in innate immunity against pathogens [86]. The present study highlights a considerable decrease in the mRNA levels of *IL-1β* and *hepcidin* among fish subjected to a stevioside-enriched diet. This indicates a significant influence of stevioside on inflammatory cytokines. Furthermore, the integration of stevioside into the diet led to a notable reduction in the expressions of *IL-1β* and *hepcidin* genes in *L. ramada*. Interestingly, a distinct down-regulation of all examined genes was evident in groups administered lower dosages (100–300 mg Stev/kg). Conversely, with increasing dosage, a dose-dependent up-regulation emerged, particularly pronounced in the 400–500 mg Stev/kg dosages.

The differential regulation of inflammatory cytokines and antimicrobial peptides in response to varying stevioside concentrations can be attributed to its dose-dependent immunomodulatory effects. At lower doses (100–300 mg Stev/kg), stevioside likely acts as an anti-inflammatory agent, potentially through the inhibition of NF-κB signaling pathways, which are crucial for the transcription of pro-inflammatory genes such as *IL-1β* [87]. This inhibition could lead to the observed down-regulation of *IL-1β* and *hepcidin*, an antimicrobial peptide also regulated by inflammatory stimuli. The reduction in hepcidin expression may additionally be linked to stevioside's potential antioxidant properties, as oxidative stress is a known inducer of hepcidin. However, at higher doses (400–500 mg Stev/kg), the dose-dependent up-regulation suggests a hormetic effect, where stevioside may act as a mild stressor, activating adaptive stress response pathways such as Nrf2-mediated antioxidant responses or mild inflammatory responses that can enhance overall immune function [87]. This biphasic response underscores the complexity of stevioside's interactions with the fish immune system and highlights the importance of dose optimization in aquaculture applications to achieve desired immunomodulatory effects without triggering excessive inflammation.

Prebiotics have demonstrated immunostimulatory effects across diverse fish species, including Caspian trout (*Salmo trutta caspius*) with β-glucan and mannan oligosaccharides (MOS) [88], Zebrafish (*Danio rerio*) utilizing galacto-oligosaccharides (GOS) [89], Common carp (*Cyprinus carpio*) incorporating GOS, fructo-oligosaccharides (FOS), and inulin [90], Nile tilapia (*Oreochromis niloticus*) responding to β-glucan [91], and European sea bass (*Dicentrarchus labrax*) with MOS [92]. Atlantic cod (*Gadus morhua*) has also shown immunostimulating effects with the use of β-glucan and MOS [93]. Further studies are essential to clarify the comparison between the findings in the current study and those of others, as the observed outcomes may vary depending on factors such as animal species, rearing conditions, age, size, trial duration, etc.

The intestine plays a crucial role in the overall well-being of fish, being intricately linked to immune response, metabolism, and the capacity to withstand environmental stress [74]. The health of the gut is paramount for animals in terms of digesting and absorbing nutrients effectively [94]. Specifically in fish, the condition of the intestines is closely tied to dietary nutrients, influencing growth, development, structural and functional aspects, as well as the processes of digestion and absorption [95]. The intestinal histological examination of *L. ramada* revealed that the intestinal wall and intestinal villi were intact in all groups examined. In the control group, the intestinal structure consisted of the tunica mucosa, composed of simple columnar enterocytes arranged in a normal arrangement, the propria submucosa, the tunica muscularis, and the outer serosa. Improved intestinal morphometry refers to the increase of the surface area of intestinal villi, which plays a key role in absorbing digested nutrients within the intestine [96]. This could be attributed to the enhanced feed efficiency which was linked to a larger surface area of intestinal villi [97]. The histological analysis revealed a dose-dependent progressive improvement in the structure of intestinal villi as the absorptive villous area increased. This improvement was most pronounced at moderate and high concentrations (300, 400, and 500 mg/kg, respectively) of stevioside. Boonkaewwan, et al. [98] observed that stevioside influenced the structure of intestinal villi in a dose-dependent manner. Stevioside and its metabolite, steviol, caused the greatest enhancement at moderate and high concentrations (300, 400, and 500 mg/kg). Stevioside and its metabolite, steviol, possess immunomodulatory and secretory properties, which contribute to the observed enhancement. Stevioside and steviol increase chloride secretion while decreasing TNF-alpha-stimulated IL-8 production, stevioside and steviol could help mitigate inflammation and promote a healthier intestinal environment. This, in turn, may support the observed changes in the structure of intestinal villi, indicating improved nutrient absorption and overall gut health.

The liver histopathological analysis across experimental groups revealed a typical spongy appearance, normal polyhedral hepatocytes, and vesicular nuclei arranged in hepatic cords around the central vein. Slight vacuolation was observed in control, low, and moderate stevioside levels. Notably, high stevioside supplementation (400, 500 µg/kg) showed a notable enhancement in hepatic architecture, accompanied by leukocytic aggregation and increased glycogen deposition. These findings suggest a potentially positive impact of high-level stevioside supplementation on liver health, indicating improved structural characteristics and immune response. This is further supported by the favorable AST and ALT indicators results, signifying overall health [19, 99]. While AST and ALT levels remained unchanged in this study, it suggests that the cold stress may not have significantly impacted liver health in juvenile thinlip mullet. The stable ALT and AST levels indicate that there was no liver damage or stress resulting from the experimental conditions. Furthermore, the stevioside supplementation appears to have helped the fish cope with cold stress, supporting their overall health and resilience. However, further immunohistochemical studies are needed to support the current study.

## Conclusion

Stevioside supplementation (300–500 mg/kg) significantly improved growth, feed efficiency, antioxidant enzymes, and immune functions in Thinlip mullet under low-temperature stress. Future studies should explore stevioside’s effectiveness under various conditions and its impact on stress responses in fish. Long-term studies and scalability evaluations are necessary for its practical application in large-scale aquaculture.

## Data Availability

The datasets underpinning this study are accessible from the corresponding author upon request.

## Abbreviations

Hep: Hepcidin
il1β: Interleukin 1-β
IBW: initial body weight
FBW: final body weight
WG: weight gain
ADG: average daily gain
SGR: specific growth rate
SR: survival rate
FI: feed intake
FCR: feed conversion ratio
GLU: glucose
TP: total protein
ALB: albumin
GLOB: globulin
T-CHOL: total cholesterol
TG: triglyceride
ALT: alanine aminotransferase
AST: aspartate aminotransferase
NBT: Nitro-blue Tetrazolium
ACH50: Serum alternative complement pathway
